# Microsatellite linkage analysis, single-nucleotide polymorphisms, and haplotype associations with ECB21 in the COGA data

**DOI:** 10.1186/1471-2156-6-S1-S94

**Published:** 2005-12-30

**Authors:** Aldi T Kraja, Ingrid B Borecki, Michael A Province

**Affiliations:** 1 Division of Biostatistics, Washington University School of Medicine, St. Louis, MO, USA

## Abstract

This study, part of the Genetic Analysis Workshop 14 (GAW14), explored real Collaborative Study on the Genetics of Alcoholism data for linkage and association mapping between genetic polymorphisms (microsatellite and single-nucleotide polymorphisms (SNPs)) and beta (16.5–20 Hz) oscillations of the brain rhythms (ecb21). The ecb21 phenotype underwent the statistical adjustments for the age of participants, and for attaining a normal distribution. A total of 1,000 subjects' available phenotypes were included in linkage analysis with microsatellite markers. Linkage analysis was performed only for chromosome 4 where a quantitative trait locus with 5.01 LOD score had been previously reported. Previous findings related this location with the *γ*-aminobutyric acid type A (GABA_A_) receptor. At the same location, our analysis showed a LOD score of 2.2. This decrease in the LOD score is the result of a drastic reduction (one-third) of the available GAW14 phenotypic data. We performed SNP and haplotype association analyses with the same phenotypic data under the linkage peak region on chromosome 4. Seven Affymetrix and two Illumina SNPs showed significant associations with ecb21 phenotype. A haplotype, a combination of SNPs TSC0044171 and TSC0551006 (the latter almost under the region of GABA_A _genes), showed a significant association with ecb21 (*p *= 0.015) and a relatively high frequency in the sample studied. Our results affirmed that the GABA region has potential of harboring genes that contribute quantitatively to the beta oscillation of the brain rhythms. The inclusion of the remaining 614 subjects, which in the GAW14 had missing data for the ecb21, can improve the strength of the associations as they have already shown that they contribute quite important information in the linkage analysis.

## Background

In the previous studies, beta (16.5–20 Hz) oscillations of the brain rhythms have been reported as being reflections of the activated states of neural networks, which are claimed to involve *γ*-aminobutyric acid type A (GABA_A_) receptor action [1]. The components (4 frequency bands: delta, theta, alpha, and beta) of the resting electroencephalogram (EEG) were found to have a large additive heritability [2]. In addition, Porjesz et al. [3] reported a 5.01 LOD score of β2 brain rhythms at about 50 cM on chromosome 4 (D4S1627) and conveyed that it represented a significant linkage and linkage disequilibrium between beta frequency and a set of GABA_A _receptor genes [3]. In this work we asked the following questions: Can the linkage results of Porjesz et al. [3] be reproduced with the data available from the Genetic Analysis Workshop 14 (GAW14)? If so, can the association tests with Affymetrix and Illumina SNPs along the same location narrow the linkage findings to potential gene(s)?

## Methods

In the GAW14 data, which made available data from the Collaborative Study on the Genetics of Alcoholism (COGA), information on 1,614 subjects was made accessible, but 614 of the subjects had missing values for ecb21 (brain rhythm beta electric oscillations). In the Porjesz et al. [3] study, the sample was drawn from 250 families and consisted of 1,553 individuals. In our analysis 354 nuclear families were considered, with 140 fathers, 190 mothers, and 826 sibs. They represented a total of 1,000 individuals and a mixture of 7 different ethnicities, with a predominance of Caucasians. In advance, the ecb21 trait was adjusted for age, and the residuals from the regression analysis were used as the final phenotype. Porjesz et al. [3] had extracted the first spatial and spectral component pair of the 5 electroencephalogram bands, where β2 demonstrated a high significant linkage. Based on instructions of GAW14 COGA data, it is believed that we are using the same trait.

Identity-by-descent (IBD) coefficients were estimated on nuclear families with the MAPMAKER/SIBS program [4]. Linkage analysis was performed as variance components linkage analysis utilizing the SEGPATH program [5]. Porjesz et al. [3] had performed IBD estimation and also variance components linkage analysis with SOLAR [6]. They also performed the tests by the multivariate t distribution, because a slight kurtosis was present in the traits. The analyses in both cases on chromosome 4 were carried out at 1-cM intervals. Our association analyses, and haplotype association tests were family-based tests, utilizing FBAT and HBAT programs [7]. Adjustments for multiple testing as a false discovery rate (FDR) adjustments on SNPs association tests were completed by using the PROC MULTTEST of SAS developed on Benjamini and Hochberg contributions, and also by using *q*-value software of Storey in R language [8,9]. The *q*-value of SNPs tests provides the proportion of false positives occurring when that particular SNP association is declared significant. The phenotype ecb21 was adjusted for age (age at the time of the clinic visit).

## Results

**Figure 1:**
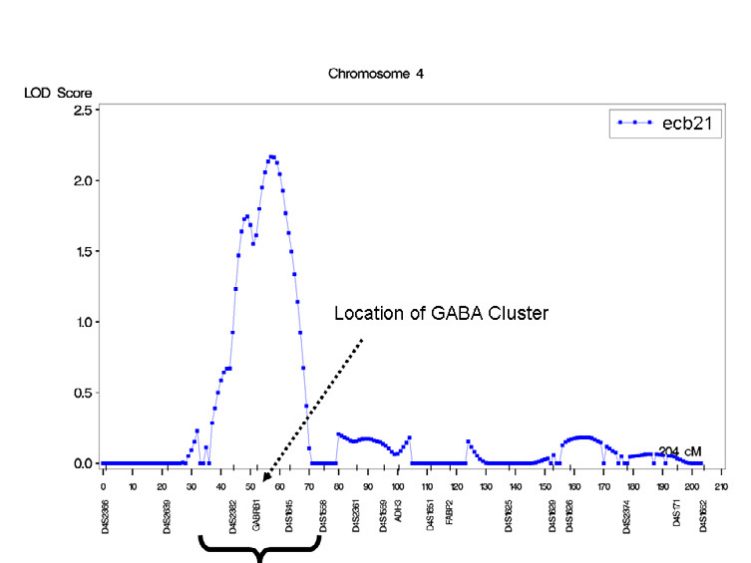
Linkage analysis of ecb21 (all data).

Porjesz et al. [3] had reported an 86% heritability for the beta spectral power. In our study, the heritability of the trait ecb21 was 68%. The linkage LOD score results reported by Porjesz et al. [3] reached a maximum LOD score of 5.01 for ecb21 at 50 cM on chromosome 4. These results were not identically replicable because the data used for the ecb21 trait was a subset of 1,000 subjects with phenotypic data, compared to 553 extra subjects used in the previous report and not present in the GAW14 data. As a result, when using these data on chromosome 4, location 57–58 cM, a reduced peak of 2.2 LOD score was found (Figure 1). Although we tested other adjustments of the trait, such as log transformation, or selected subsets of data based on ethnicity, the LOD scores did not improve (results not shown). After communicating with the corresponding author of the original study [3], it was found that lower LOD scores compared to the previous linkage findings were mainly the result of missing (for objective reasons) one-third of the original sample (Porjesz, personal communication). Therefore, we decided to use the GAW14 ecb21 trait for all the data adjusted for age, as the phenotype for any other analysis.

**Figure 2:**
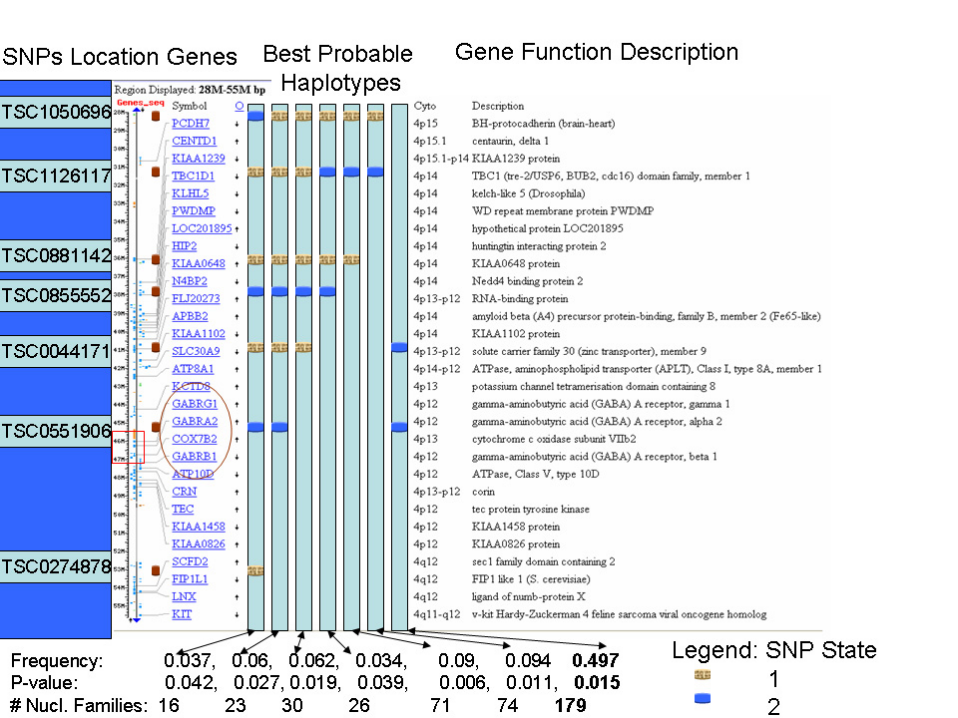
**Significant associated haplotypes, significant SNP locations, and genes**. Left, the names of 7 Affymetrix SNPs. They are followed by two vertical lines, ticks of one of them represent the physical map in Mb. The next column lists gene symbols. The GABA region is circled. Seven columns follow, which represent best haplotypes and their color-coded SNP states. The next column represents the cytogenetic locations, which are followed by gene function descriptions. At the bottom of the figure, the frequency, *p*-value of the association of the best haplotype to the ecb21, and the number of nuclear families a particular haplotype is present, are provided.

We restricted the association tests to the region of the linkage signal (39 cM = dist = 70 cM) in order to decrease the possibilities of having false-positive associations (Figure 2). Employing FBAT under the assumption of linkage and computing test statistics using the empirical variance for each SNP, 102 Affymetrix SNPs and 34 Illumina SNPs were evaluated. Three models (additive, recessive, and dominant) were assessed. A total of 9 SNPs showed nominally significant associations under different models, of which 7 originated from the Affymetrix set (TSC1050696, TSC1126117, TSC0881142, TSC0855552, TSC0044171, TSC0551906, and TSC0274878) and 2 from the Illumina set (rs790142, and rs140643). After adjustments for multiple comparisons (two methods applied in the whole markers set studied), none of the 9 association tests remained significant (results not shown). It is known that haplotype association analysis has more power than single association tests of SNPs. Due to time constraints, we performed haplotype analysis for only the 7 Affymetrix SNPs that already had a nominal significant association with ecb21 (Figure 2). A haplotype of two SNPs (TSC0044171 and TSC0551906) was quite common in the population and associated significantly with the ecb21.

## Discussion

The heritability of the trait studied was high (68 ± 6%). We found evidence of linkage for ecb21 at the published location. The LOD score we observed using the available data was lower than the previous published value. There were two possible peaks, with the main one located within 57–58 cM (Figure 1). The candidate for the linkage location identified by Porjesz et al. [3] is the gamma-aminobutyric acid (GABA_A_) receptor, which is a multisubunit chloride channel that mediates the fastest inhibitory synaptic transmission in the central nervous system. It is located at 4p12, in the physical chromosomal interval 46.6–47.5 Mb, and at 51.4 cM based on GAW14 genetic map. GABA_A _is contained in a cluster consisting of genes encoding alpha 4, alpha 2, and gamma 1 subunits of the GABA_A _receptor. Modifications of this gene were implicated in the pathogenetics of schizophrenia [3,10].

The associations of SNPs with ecb21 within this region were tested. Out of the nine nominally significant SNPs (7 for Affymetrix SNPs AND 2 for Illumina), none of them hit the location 46.6–47.5 Mb, but some of them were very close to the GABA_A _receptor gene, and the surrounding region (Figure 2). These SNPs are located under the linkage peak, which made these findings exciting. The application of the FDR adjustments for multiple tests showed that all the association tests became non-significant. This leaves open the question whether the significant associations were true associations or not. It is our impression that our FDR *p*-value adjustments were conservative, because we corrected *p*-values for the multiple testing for the whole region. Therefore, we think that haplotype analysis can be more useful because it reduces the dimensionality of the tests. Different marker sliding-windows combinations of the 7 nominally significant Affymetrix SNPs were performed. The best significant haplotypes per window in association with ecb21 are reported (Figure 2). We were not able to convey any important biological meaning of different significant haplotypes. A combination of two SNPs, TSC0044171 and TSC0551006 (the latter almost under the region of GABA_A _genes), showed a significant association with ecb21 (*p *= 0.015) and a relatively high frequency in the sample studied. The inclusion of the remaining 614 subjects, that in the GAW14 have missing data for the ecb21, can improve the strength of the associations as they have shown already that they contribute quite important information in the linkage analysis (previous finding of a peak of 5.01 LOD score [3]).

## Conclusion

The previously reported linkage peak of 5.01 for ecb21 located on chromosome 4, was reproduced by this analysis, but attenuated to a 2.2 LOD score as a result of unavailable phenotypes for 614 subjects. With the reduced sample of 1,000 subjects, 9 SNPs, 7 of Affymetrix and 2 of Illumina, showed nominally significant associations with ecb21. A common haplotype of 2 SNPs states showed a significant association with ecb21. The presence of these findings around the GABA cluster supports the formerly proposed relation among this cluster, brain oscillations, and alcoholism. The inclusion of the other 614 subjects in the analysis may strengthen the association signals, and provide better evidence on the GABA cluster role, as it has been shown that these extra subjects carry important information in the linkage analysis.

## Abbreviations

COGA: Collaborative Study on the Genetics of Alcoholism

EEG: Electroencephalogram

FDR: False discovery rate

GABA_A_: Gamma-aminobutyric acid

GAW14: Genetic Analysis Workshop 14

IBD: Identity by descent

QTL: Quantitative trait loci

SNP: Single-nucleotide polymorphism
